# The NRF2 transcriptional target, *OSGIN1*, contributes to monomethyl fumarate-mediated cytoprotection in human astrocytes

**DOI:** 10.1038/srep42054

**Published:** 2017-02-09

**Authors:** Melanie S. Brennan, Maria F. Matos, Karl E. Richter, Bing Li, Robert H. Scannevin

**Affiliations:** 1 Neurology Research, Biogen Inc., Cambridge, MA, 02142, USA; 2 Boston University School of Medicine, Boston, MA 02118, USA.

## Abstract

Dimethyl fumarate (DMF) is indicated for the treatment of relapsing multiple sclerosis and may exert therapeutic effects via activation of the nuclear factor (erythroid-derived 2)-like 2 (NRF2) pathway. Following oral DMF administration, central nervous system (CNS) tissue is predominantly exposed to monomethyl fumarate (MMF), the bioactive metabolite of DMF, which can stabilize NRF2 and induce antioxidant gene expression; however, the detailed NRF2-dependent mechanisms modulated by MMF that lead to cytoprotection are unknown. Our data identify a mechanism for MMF-mediated cytoprotection in human astrocytes that functions in an OSGIN1-dependent manner, specifically via upregulation of the OSGIN1-61 kDa isoform. NRF2-dependent OSGIN1 expression induced P53 nuclear translocation following MMF administration, leading to cell-cycle inhibition and cell protection against oxidative challenge. This study provides mechanistic insight into MMF-mediated cytoprotection via NRF2, OSGIN1, and P53 in human CNS-derived cells and contributes to our understanding of how DMF may act clinically to ameliorate pathological processes in neurodegenerative disease.

Reactive oxygen species (ROS) and electrophiles are essential for cellular functions, including signaling, immune responses and metabolic processes^1^,^2^,^3^. However, if not homeostatically maintained, ROS and electrophiles can cause cellular damage^4^, which has been mechanistically linked to neurodegenerative disease pathology in Alzheimer’s, Parkinson’s and Huntington’s diseases, amyotrophic lateral sclerosis and multiple sclerosis (MS)^5^. When challenged with inflammatory, oxidative, or electrophilic stress, the primary mechanism for maintenance of cellular redox balance is the nuclear factor (erythroid-derived 2)-like 2 (NRF2) pathway^6^. NRF2 activation initiates gene transcription to upregulate cellular processes involved with neutralization and removal of toxic cell stressors^7^,^8^.

Under physiological conditions, NRF2 is sequestered in the cytoplasm by Kelch-like ECH-associated protein-1 (KEAP1), which constitutively targets NRF2 for ubiquitination and proteosomal degradation^9^. Modifications of KEAP1 cysteine residues via ROS/electrophiles reduce the affinity of KEAP1 for NRF2 culminating in NRF2 liberation^10^. Nuclear translocation of unbound NRF2 initiates transcription of anti-inflammatory and cytoprotective genes^7^. NRF2 activation can be induced by naturally-occurring and synthetic compounds^11^, including dimethyl fumarate (DMF)^12^ which is approved for the treatment of relapsing forms of MS^13^.

DMF and its bioactive metabolite, monomethyl fumarate (MMF), have demonstrated NRF2 stabilization and increased NRF2 target-gene expression including nicotinamide adenine dinucleotide phosphate dehydrogenase, quinone 1 (*NQO1*) and heme oxygenase-1 (*HMOX-1*)^14^,^15^,^16^,^17^. Furthermore, *in vitro* addition of DMF or MMF resulted in KEAP1 cysteine modifications, confirming these compounds activate NRF2 similarly to ROS/electrophiles^12^,^15^. Fumarates also protect primary central nervous system (CNS) cells against oxidative insults, an effect lost in the absence of NRF2^16^. In an experimental MS model, DMF increased NRF2 levels in the CNS, which correlated with symptom amelioration and preservation of myelin and neurons^12^. These effects were lost in NRF2-deficient mice. These studies support NRF2-pathway regulation as an important mechanism of action for fumarates, especially for neuroprotection. However, beyond modulation of NRF2 and antioxidant gene regulation, fumarate-induced, NRF2-dependent mechanisms of cellular protection are unknown.

*In vivo* mouse transcriptional studies uncovered diverse tissue-dependent NRF2 target genes following DMF treatment, particularly in the CNS, including oxidative stress induced growth inhibitor 1 (*OSGIN1*), also known as ovary, kidney and liver protein 38 (OKL38) or bone marrow stromal cell-derived growth inhibitor (BDGI)^17^. Although one study identified *OSGIN1* as an NRF2 transcriptional target^18^, the majority of *OSGIN1* research describes this gene under the transcriptional control of tumor suppressor protein 53 (P53) in mediating cell growth, differentiation and death^19^,^20^. These studies used tumor cells and suggested alternative splicing of *OSGIN1* contributed to its function^21^. However, it is unknown whether *OSGIN1* and its isoforms are differentially regulated across cell types or if transcription factors differentially regulate *OSGIN1* isoforms. The aim of this study was to evaluate *OSGIN1* transcription using CNS-specific cells and investigate *OSGIN1* in MMF-mediated cytoprotection.

Our study uncovers previously unknown functions of NRF2 and validates that in human astrocytes, *OSGIN1* is under the transcriptional control of NRF2 rather than P53. In contrast to studies using tumor cells, we identify a novel mechanism by which *OSGIN1* mediates astrocyte protection against hydrogen peroxide (H_2_O_2_)-induced oxidative injury via induction and nuclear translocation of P53. Finally, we show that *OSGIN1* cooperates with NRF2 and P53 to protect astrocytes against oxidative insult in the presence of MMF.

## Results

### MMF-dependent *OSGIN1* induction is modulated via NRF2

We previously described DMF *in vivo* administration to mice induced NRF2-dependent gene transcription throughout the brain, and *Osgin1* was the most robustly modulated transcript^17^. To better understand the significance of *OSGIN1* as an NRF2- and DMF-dependent target gene, *OSGIN1* transcript modulation following MMF treatment in primary cultures of human spinal cord astrocytes was investigated. Because DMF is rapidly hydrolyzed to MMF following oral administration and the CNS is predominantly exposed to MMF after oral DMF dosing^17^,^22^, studies were conducted using MMF. Human astrocytes were chosen for *in vitro* investigation of *OSGIN1* regulation because the NRF2 pathway is relatively dormant in adult neurons but strongly modulated in astrocytes^23^,^24^, and astrocyte overexpression of NRF2 confers protection to neurons in astrocyte-neuronal co-cultures^25^.

**Figure 1:**
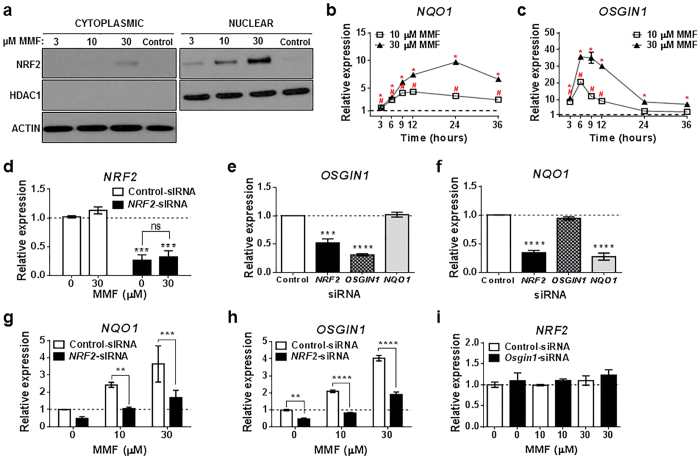
MMF induces NRF2 nuclear translocation and NRF2-dependent transcription of *OSGIN1* mRNA in human spinal cord astrocytes. (**a**) Western blot of NRF2 from astrocyte protein extracts following 0 (Control), 3, 10, or 30 μM MMF treatment for 6 hours. HDAC1 and ACTIN were used as loading controls and to specify nuclear versus cytoplasmic expression, respectively. (**b–i**) Transcript analyses of RNA extracts from astrocytes using qRT-PCR. Data are graphed on the Y-axis as relative expression of gene modulation relative to controls: time-matched DMSO controls (**b**,**c**), control-siRNA with 0 μM MMF (**d**,**g**–**i**) or control-siRNA (**e**,**f**). Gene expression was normalized to 1 (dashed line). (**b**,**c**) qRT-PCR of *NQO1* (**b**) and *OSGIN1* (**c**) mRNA following 10 or 30 μM MMF treatment after 3, 6, 9, 12, 24, or 36 hours (*n* = 2/condition/time point). *p* < 0.05 (unpaired *t* test) for 10 μM MMF (#) and 30 μM MMF (*). (**d**) qRT-PCR for *NRF2* mRNA following transfection with control- or *NRF2*-siRNA and hour treatment with 0 or 30 μM MMF. Mean ± SEM shown for 3 independently performed experiments with *n* = 2/condition. ***p < 0.001 (two-way ANOVA with Tukey’s multiple comparisons) compared with control-siRNA with 0 μM MMF. ns = not significant. (**e**,**f**) qRT-PCR analysis for *OSGIN1* (**e**) and *NQO1* (**f**) mRNA following transfection with control-, *NRF2*-, *OSGIN1*-, or *NQO1*-siRNA. Mean ± SEM shown for 3 independently performed experiments with *n* = 2/condition. ***p < 0.001 and ****p < 0.0001 (one-way ANOVA with Tukey’s multiple comparisons) compared with control-siRNA. (**g**,**h**) qRT-PCR for *NQO1* (**g**) and *OSGIN1* (**h**) mRNA following transfection with control- or *NRF2*-siRNA. Mean ± SD shown for *n* = 4/condition. **p < 0.01, ***p < 0.001 and ****p < 0.0001 (two-way ANOVA with Tukey’s multiple comparisons). (**i**) qRT-PCR for *NRF2* mRNA following transfection with control- or *OSGIN1*-siRNA. Mean ± SD shown for *n* = 4/condition.

To confirm NRF2 protein accumulation in human astrocytes following MMF treatment, cells were exposed to MMF for 6 hours and NRF2 expression assessed in cytoplasmic and nuclear fractions by Western immunoblot (Fig. 1a). Nuclear and cytoplasmic specificity was confirmed using the nuclear-specific protein, HDAC1. Consistent with previous reports^16^, MMF induced dose-dependent NRF2 accumulation predominantly within the nuclear fraction (Fig. 1a). To determine whether NRF2 nuclear localization following MMF treatment resulted in NRF2-dependent gene transcription, the classical NRF2 target gene, *NQO1*, and our previously identified NRF2 target gene, *OSGIN1*, were assessed via quantitative real-time polymerase chain reaction (qRT-PCR) following MMF treatment in astrocytes (Fig. 1b,c). MMF administration resulted in significant (*p* < 0.05) induction of *NQO1* (Fig. 1b) and *OSGIN1* (Fig. 1c) in a time and dose-dependent manner, with *OSGIN1* peak expression reaching ~35-fold above baseline. Consistent with our previous findings in mouse, MMF treatment induced transient *OSGIN1* expression which peaked at early time points (6 to 9 hours post-compound addition) compared with *NQO1*, which peaked around 24 hours with prolonged expression^17^. This divergence in expression patterns suggests various functions associated with the NRF2 pathway.

Our previous work in mouse models demonstrated that *Osgin1* transcript induction following DMF administration was NRF2-dependent^17^. In primary human astrocyte cultures using *NRF2*- and *OSGIN1*-specific siRNA to reduce mRNA levels, *OSGIN1* expression following MMF treatment was NRF2-dependent. Transfection with *NRF2*-siRNA resulted in ~75% loss of total *NRF2* transcript expression (*p* = 0.0006) compared with control-siRNA, with no significant induction with 30 μM MMF (Fig. 1d). Because NRF2 is constitutively expressed and degraded under normal homeostatic conditions by KEAP1, and inhibition of this interaction is believed to regulate NRF2 protein expression, no change in *NRF2* mRNA induction was expected following MMF treatment^9^. *NRF2* knockdown decreased *OSGIN1* mRNA expression by ~50% (*p* = 0.0002) compared with control-siRNA, and *OSGIN1* knockdown resulted in ~70% decrease in *OSGIN1* levels (*p* < 0.0001) (Fig. 1e). Using *NQO1*-siRNA as a control, decreased *NQO1* mRNA levels had no effect on *OSGIN1* mRNA (Fig. 1e). *NQO1* mRNA was also probed following siRNA knockdown of *NRF2, OSGIN1*, and *NQO1*. Significant reductions in *NQO1* transcript levels were detected following cellular transfection with *NRF2*- and *NQO1*-siRNA (*p* < 0.0001), with no change in *NQO1* levels following *OSGIN1*-siRNA transfection (Fig. 1f). *NRF2* knockdown also significantly prevented MMF-dependent increases in *NQO1 (p* = 0.0064, 10 μM; *p* = 0.0002, 30 μM) and *OSGIN1 (p* = 0.0079, 0 μM; *p* < 0.0001, 10 μM; *p* < 0.0001, 30 μM) mRNA expression (Fig. 1g,h). Furthermore, *OSGIN1* knockdown had no effect on *NRF2* transcript expression (Fig. 1i). These findings demonstrate that *OSGIN1* is induced downstream of NRF2 following MMF administration in primary human astrocyte cultures similar to our findings in mouse tissues after *in vivo* DMF administration^17^.

### *OSGIN1* transcript expression is modulated independently of P53 in human astrocytes

**Figure 2:**
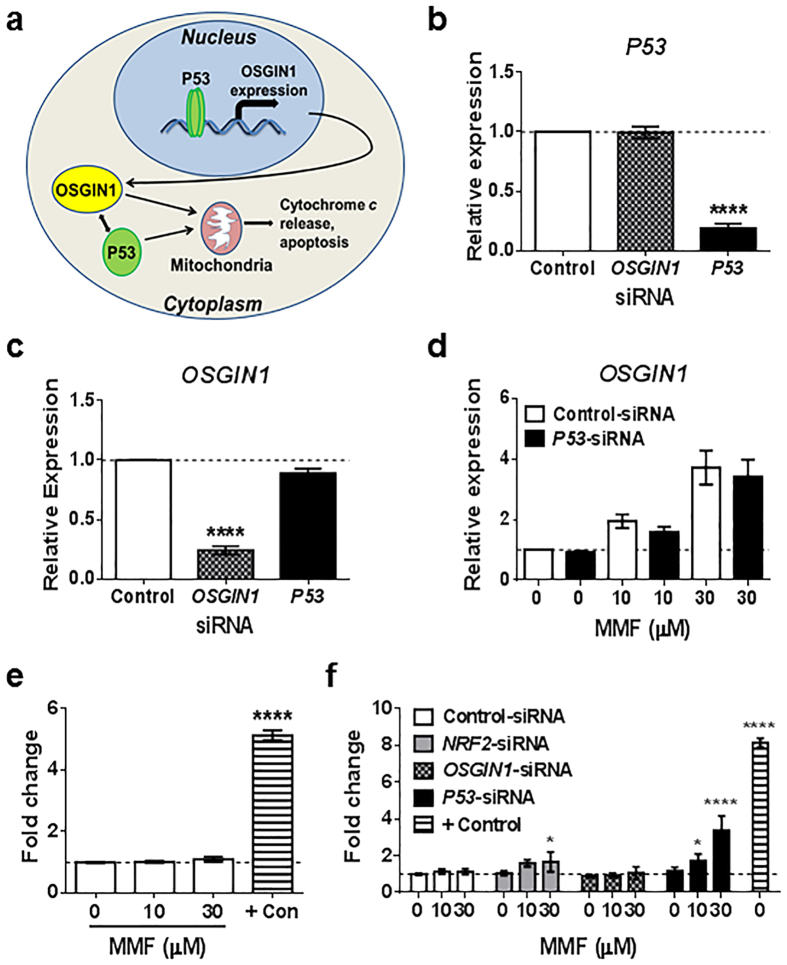
*OSGIN1* is not transcriptionally regulated by P53 and does not induce cellular apoptosis in human spinal cord astrocytes. (**a**) Schematic illustration of *OSGIN1* under the transcriptional control of P53 in immortalized/transformed cells. P53 binds to the promoter region of *OSGIN1* to induce transcription. Translated OSGIN1 protein physically interacts with P53 protein to induce cytochrome c release in mitochondria and subsequent apoptosis. (**b**–**d**) qRT-PCR of astrocyte RNA extracts. Data graphed as relative expression of gene modulation relative to control-siRNA (**b**,**c**) or control-siRNA with 0 μM MMF (**d**). Gene expression was normalized to 1 (dashed line). (**b**,**c**) qRT-PCR for *P53* (**b**) and *OSGIN1* (**c**) mRNA following transfection with control-, *OSGIN1*-, or *P53*-siRNA. Mean ± SEM shown for 4 independently performed experiments with *n* = 2 to 4/condition. ****p < 0.0001 (one-way ANOVA with Dunnett’s multiple comparisons) compared with control-siRNA. (**d**) qRT-PCR for *OSGIN1* mRNA following transfection with control- or *P53*-siRNA. Mean ± SEM shown for 3 independently performed experiments with *n* = 2/condition. **p < 0.01 (one-way ANOVA with Tukey’s multiple comparisons) compared with control-siRNA. (**e**,**f**) Quantification of cellular apoptosis using TiterTACS™ in human spinal cord astrocytes. Data graphed as fold change relative to 0 μM MMF and normalized to 1 (dashed line). Nuclease-treated cells were included as a positive control (+Con). (**e**) Quantification of apoptosis following 0, 10, or 30 μM MMF. *n* = 4/condition. ****p < 0.0001 (one-way ANOVA with Tukey’s multiple comparisons) compared with 0 μM MMF. (**f**) Quantification of apoptosis following transfection with control-, *NRF2*-, *OSGIN1*-, or *P53*-siRNA and treated with 0, 10, or 30 μM MMF. *n* = 6/condition. *p < 0.05 and ****p < 0.0001 (two-way ANOVA with Tukey’s multiple comparisons) compared with control-siRNA treated with 0 μM MMF.

Recent tumorigenesis studies have identified OSGIN1 as an apoptotic regulator under the transcriptional control of P53^26^. OSGIN1 was found to co-localize with P53 in mitochondria to induce cytochrome C release, suggesting P53 transcriptionally regulates *OSGIN1* as well as mediates its protein function (Fig. 2a). However, our research in human astrocytes suggests *OSGIN1* is under the transcriptional control of NRF2 (Fig. 1e,h). To determine whether *OSGIN1* transcription is P53-dependent in a non-transformed cell model, *OSGIN1* and *P53* mRNA were decreased in primary human spinal cord astrocytes using *OSGIN1*- and *P53*-siRNA. Transfection of astrocytes with *P53*-siRNA resulted in ~80% reduction in *P53* mRNA compared with control-siRNA (*p* < 0.0001), whereas *OSGIN1* knockdown had no effect on *P53* mRNA levels (Fig. 2b). P53 knockdown yielded no change in *OSGIN1* mRNA levels in contrast to ~75% reduction of *OSGIN1* mRNA following *OSGIN1*-siRNA transfection (*p* < 0.0001) (Fig. 2c). Loss of P53 did not affect MMF (10 and 30 μM) from significantly increasing *OSGIN1* transcript levels in astrocytes (Fig. 2d). Combined with our findings that *OSGIN1* is downstream of NRF2 (Fig. 1), *OSGIN1* is a transcriptional target of NRF2 in human astrocytes and is not under the transcriptional control of P53.

To determine whether NRF2-dependent *OSGIN1* expression induces apoptosis similar to findings in transformed tumor cells^26^, astrocytes were treated with 10 or 30 μM of MMF for 48 hours followed by *in situ* labeling of DNA fragmentation as a measure of apoptosis. MMF administration did not significantly induce apoptosis compared with untreated cells (Fig. 2e). Nuclease-treated cells were included as a positive control and to verify assay performance (Fig. 2e). To identify potential effects of reduced *NRF2, OSGIN1*, and *P53* expression on apoptosis of astrocytes, *in situ* labeling of DNA fragments was analyzed in cells transfected with *NRF2*-, *OSGIN1*-, and *P53*-siRNA and treated with 10 or 30 μM MMF. Compared with control-siRNA, *NRF2*-, *OSGIN1*-, and *P53*-siRNA transfection did not significantly induce apoptosis; however, in the presence of MMF, *NRF2*- (*p* = 0.0375, 30 μM) and *P53*-siRNA (*p* = 0.0186, 10 μM; *p* < 0.0001, 30 μM) transfection significantly induced apoptosis compared with untreated control groups (Fig. 2f). In contrast, *OSGIN1* knockdown in the presence of increasing MMF concentrations did not induce significant apoptosis in these cells (Fig. 2f). These results indicate MMF activation of the NRF2 pathway, which induces *OSGIN1* transcript, does not promote apoptosis in human astrocyte cultures. Therefore, *OSGIN1* under the control of NRF2 in primary astrocytes does not modulate apoptosis in contrast to *OSGIN1* under the control of P53 in immortalized cells. Furthermore, MMF induction of apoptosis following *NRF2* or *P53* knockdown suggests these transcription factors have complex functions and cells may become hypersensitive to electrophilic challenge in their absence.

### NRF2 and OSGIN1 contribute to MMF-mediated cytoprotection

**Figure 3:**
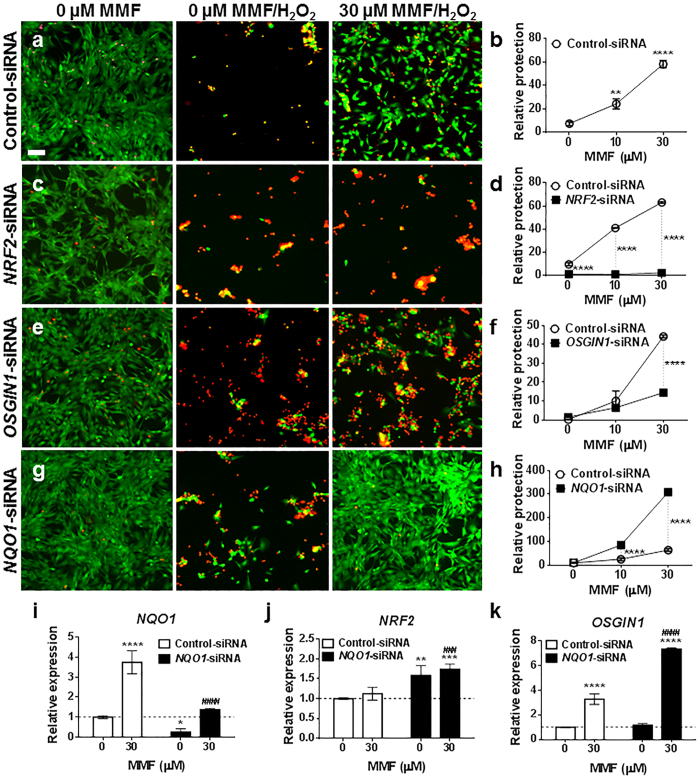
*NRF2* and *OSGIN1* contribute to MMF-mediated cytoprotection after oxidative challenge in human spinal cord astrocytes. (**a**,**c**,**e**,**g**) Astrocytes transfected with control- (**a**), *NRF2*- (**c**), *OSGIN1*- (**e**) or *NQO1*-siRNA (**g**) were pretreated with 0, 10, or 30 μM MMF and then challenged with 200 μM H_2_O_2_ followed by a 20 hour recovery. Live imaging was used to differentiate live (calcein AM labeling, green) vs dead (ethidium homodimer labeling, red) cells. Representative images have identical histogram lookup tables for display comparison. Scale bar: 0.03 mm. Data are repeated across 3 independent experiments. (**b**,**d**,**f**,**h**) Replicate plates as in (**a**,**c**,**e**,**g**) were fixed and stained with DAPI. Relative protection is graphed on the Y-axis as mean ± SD for cell nuclei counts from duplicate wells (*n* = 15 fields per well/condition). (**b**) **p = 0.0028 and ****p < 0.0001 compared with 0 μM MMF (one-way ANOVA with Dunnett’s multiple comparisons). (**d**,**f**,**h**) ****p < 0.0001 compared with control-siRNA treated with 0 μM MMF (two-way ANOVA with Sidak’s multiple comparisons). Data repeated across 3 independent experiments. (**i**–**k**) qRT-PCR for *NQO1* (**i**), *NRF2* (**j**), and *OSGIN1* (**k**) mRNA expression following transfection with control- or *NQO1*-siRNA and treated with 0 or 30 μM MMF. Data graphed as relative expression of gene modulation relative to control-siRNA treated with 0 μM MMF and normalized to 1 (dashed line). Mean ± SD shown for *n* = 4/condition. *p < 0.05, **p < 0.01, and ****p < 0.0001 (two-way ANOVA with Tukey’s multiple comparisons) compared with control-siRNA treated with 0 μM MMF. ^###^*p* < 0.001 and ^####^*p* < 0.0001 (two-way ANOVA with Tukey’s multiple comparisons) compared with control-siRNA treated with 30 μM MMF.

Having determined MMF-induced *OSGIN1* transcript expression was NRF2-dependent (Fig. 1e,h), we next examined *OSGIN1* contribution to MMF-mediated cytoprotection in astrocytes transfected with *NRF2*- or *OSGIN1*-siRNA. Because the NRF2 pathway is considered the primary cellular defense against oxidative and electrophilic stress^6^, transfected astrocytes were treated with MMF for 24 hours to activate the NRF2 pathway followed by H_2_O_2_-induced oxidative insult. Relative protection was quantitated post-treatment using immunofluorescence to label live cells with calcein-AM (green) and dead cells with ethidium homodimer (red) (Fig. 3a,c,e,g). Immunofluorescence results were confirmed using viable nuclear count with DAPI nuclear stain (Fig. 3b,d,f,h). Virtually no live cells were detected in control-siRNA transfected cultures following H_2_O_2_ insult unlike dimethyl sulfoxide (DMSO)-treated cells (Fig. 3a). In the presence of 30 μM MMF, cells were protected from H_2_O_2_ toxicity by ~60% (*p* < 0.0001) (Fig. 3a,b). In contrast to cultures transfected with control-siRNA, MMF did not protect cells transfected with *NRF2*-siRNA and treated with H_2_O_2_ (*p* < 0.0001), confirming the importance of NRF2 in MMF-mediated cytoprotection of astrocytes (Fig. 3c,d). Similar to *NRF2* knockdown, *OSGIN1-*siRNA knockdown significantly (*p* < 0.0001) reduced MMF-dependent protection of astrocytes against toxic H_2_O_2_ (Fig. 3e,f). Unlike *NRF2* knockdown, *OSGIN1* knockdown did not completely abolish MMF-mediated cytoprotection, with ~10% of protection maintained at 30 μM MMF (Fig. 3f). Although this could be a result of incomplete *OSGIN1* loss, other NRF2 transcriptional targets may contribute to MMF-mediated protection. These results support a protective role for NRF2-dependent *OSGIN1* expression following MMF treatment in human astrocytes.

To determine whether additional NRF2 transcriptional targets contribute to MMF-mediated cytoprotection, siRNA knockdown of the classical NRF2 target gene *NQO1* was assessed in H_2_O_2_-treated astrocytes. As described earlier, *NQO1* was induced following MMF administration in astrocytes in an NRF2-depedenent manner (Fig. 1b,g), similar to *OSGIN1* (Fig. 1c,h). In contrast to *OSGIN1*-siRNA knockdown, loss of *NQO1* did not abrogate MMF-mediated cytoprotection in astrocytes following H_2_O_2_ insult (Fig. 3g,h). Instead, *NQO1* mRNA reductions resulted in significantly greater protection (*p* < 0.0001) against H_2_O_2_ in the presence of MMF compared with control-siRNA. Furthermore, *NQO1* knockdown significantly induced *NRF2* mRNA (*p* = 0.0011, 0 μM; *p* = 0.0001, 30 μM) compared with untreated control-siRNA; however, no additional *NRF2* mRNA induction was identified following *NQO1* knockdown in the presence of MMF (Fig. 3i,j). Regarding *OSGIN1* expression, *NQO1* knockdown significantly (*p* < 0.0001) increased *OSGIN1* compared with control-siRNA following MMF addition but not in the absence of MMF (Fig. 3k). These findings suggest MMF induces *OSGIN1* more robustly following *NQO1* knockdown leading to increased protection against H_2_O_2_, which correlates with the protective characteristics of *OSGIN1* seen in H_2_O_2_-treated astrocytes (Fig. 3e,f).

### MMF induces the 61 kDa isoform of OSGIN1

**Figure 4:**
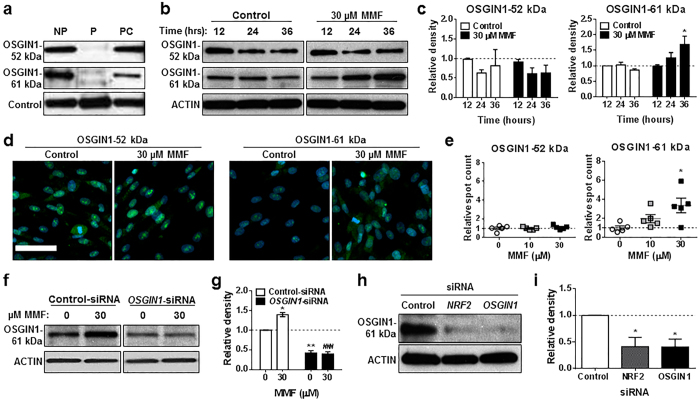
MMF induces the OSGIN1-61 kDa isoform in human astrocytes in an NRF2-dependent manner. (**a**) To confirm antibody specificity, OSGIN1-52 and −61 kDa antibodies were pre-incubated with isoform-specific (P) and nonspecific peptides (PC) followed by Western blotting of astrocyte extracts. NP = no peptide; control = non-specific reactive band. (**b**) Western blot of OSGIN1-52 and −61 kDa from astrocyte extracts following 0 (Control) or 30 μM MMF for 12, 24, or 36 hours. ACTIN = loading control. (**c**) Quantification of **b** using densitometry. Data graphed as relative density compared to control at 12 hours and normalized to 1 (dashed line). Mean ± SEM for 3 independently performed experiments. *p = 0.013 (two-way ANOVA with Sidak’s multiple comparisons) compared with time-matched control. (**d**) Representative astrocyte images labeled with OSGIN1-52 and −61 kDa antibodies following 24-hour treatment with 0, 10, or 30 μM MMF. (**e**) Quantification of immunoreactive puncta in **d**. Data graphed as relative puncta (mean ± SD from 5 wells (n = 30 fields/well/condition)) compared to 0 μM MMF and normalized to 1 (dashed line). *p = 0.0144 (one-way ANOVA with Dunnett’s multiple comparisons) compared with 0 μM MMF. (**f**) Western blot of OSGIN1-61 kDa from astrocyte extracts following control- or *OSGIN1*-siRNA transfection and treated with 0 or 30 μM MMF for 24 hours. ACTIN = loading control. (**g**) Quantification of **f** using densitometry. Data graphed as relative density compared to control-siRNA treated with 0 μM MMF and normalized to 1 (dashed line). Mean ± SEM for 2 independent experiments. *p = 0.0146 and **p = 0.0035 (two-way ANOVA with Tukey’s multiple comparisons test) compared with 0 μM MMF. ^###^*p* = 0.0004 (two-way ANOVA with Tukey’s multiple comparisons) compared with 30 μM MMF. (**h**) Western blot of OSGIN1-61 kDa from astrocyte extracts following control-, *NRF2*-, or *OSGIN1*-siRNA transfection. ACTIN = loading control. (**i**) Quantification of **h** using densitometry. Data graphed as relative density compared to control-siRNA and normalized to 1 (dashed line). Mean ± SEM for 2 independently performed experiments. *p < 0.05 (one-way ANOVA with Dunnett’s multiple comparisons) compared with control-siRNA.

The human *OSGIN1* gene undergoes alternative splicing to yield isoforms with divergent biological functions^27^,^28^. Regulation of OSGIN1 isoforms was analyzed following treatment of astrocytes with 30 μM MMF for 12, 24, or 36 hours (Fig. 4b,c). Antibodies were generated for the common human OSGIN1 isoforms and specificity was confirmed for both the OSGIN1-52 kDa and −61 kDa reactive antibodies (Fig. 4a). Immunoblots of MMF-treated astrocytes were probed with OSGIN1-52 and −61 kDa antibodies. OSGIN1-61 kDa was robustly increased in an MMF concentration-dependent manner with significance at 36 hours (*p* = 0.013), whereas OSGIN1-52 kDa remained unchanged (Fig. 4b,c). Findings were confirmed using immunofluorescence microscopy. Astrocytes were exposed to 10 or 30 μM MMF for 24 hours and assessed for OSGIN1-52 and −61 kDa isoform expression using high-content analysis to count individual immunoreactive puncta as “spots” (Fig. 4d,e). Relative spot counts of OSGIN1-52 kDa protein were unchanged following MMF treatment compared with a dose-dependent increase in OSGIN1-61 kDa protein spot counts within the cytoplasm, with significant induction seen with 30 μM MMF (*p* = 0.0144) (Fig. 4e). To confirm immunoreactive bands were OSGIN1-specific, astrocytes were transfected with *OSGIN1*-siRNA followed by treatment with 30 μM MMF for 24 hours. *OSGIN1* knockdown resulted in a significant loss of the MMF-induced OSGIN1-61 kDa immunoreactive band compared with astrocytes transfected with control-siRNA (*p* = 0.0004) (Fig. 4f,g). These findings suggest that MMF addition to astrocytes specifically induces expression of the 61 kDa OSGIN1 isoform.

Overexpression of the OSGIN1-61 kDa isoform has been shown to be less toxic to tumor cell lines compared to overexpression of the OSGIN1-52 kDa and shorter isoforms, suggesting this longer variant may function independently of apoptotic induction and thus be differentially regulated^21^,^28^. We demonstrated that *OSGIN1* expression in human astrocytes was not associated with apoptosis (Fig. 2e,f) and was NRF2-dependent (Fig. 1e,h). Therefore, OSGIN1-61 kDa regulation was examined following NRF2 depletion. Astrocytes were transfected with control-, *NRF2*- or *OSGIN1*-siRNA and cell lysates probed with OSGIN1-61 kDa antibody. NRF2 knockdown resulted in significant depletion of OSGIN1-61 kDa immunoreactivity (*p* = 0.0373), which correlated with *OSGIN1*-siRNA knockdown (*p* = 0.0240) (Fig. 4h,i), confirming regulation of this isoform was NRF2-dependent. These results identify the full-length 61 kDa OSGIN1 isoform is modulated downstream of NRF2 following MMF administration in human astrocytes.

### MMF induces alterations in the 5′ UTR of the *OSGIN1* transcript

**Figure 5:**
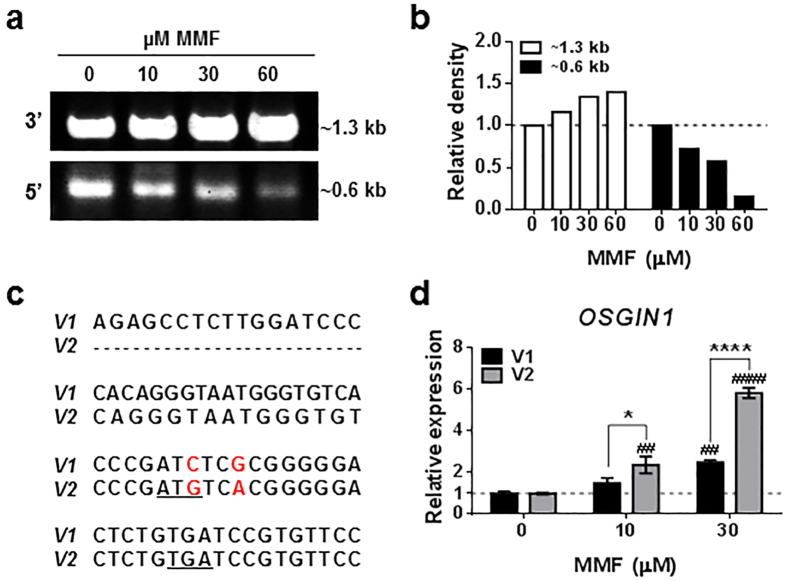
MMF induces alterations in the 5′ UTR of *OSGIN1* in human spinal cord astrocytes. (**a**) Agarose gel electrophoresis of 3′ and 5′ RACE products of *OSGIN1* following 0, 10, 30, or 60 μM MMF in astrocytes. (**b**) Quantification of **a** using densitometry. Data graphed as relative density compared to 0 μM MMF and normalized to 1 (dashed line). 3′ and 5′ RACE data are normalized to their respective 0 μM MMF values. (**c**) DNA sequencing results of 3′ and 5′ RACE products from **a** identifying two alternate variants of *OSGIN1*, V1 and V2. Underlined letters indicate potential start (ATG) and stop (TGA) codons identified in the V2 variant following nucleotide substitutions indicated in red. (**d**) qRT-PCR of *OSGIN1* transcript variants identified in **c** (V1 and V2) following treatment of human astrocytes with 0, 10, or 30 μM MMF. Data graphed as relative expression of gene modulation relative to individual variants (V1 or V2) treated with 0 μM MMF and normalized to 1 (dashed line). Mean ± SD shown for *n* = 2/condition. ^##^*p* < 0.01 and ^####^*p* < 0.0001 (two-way ANOVA with Tukey’s multiple comparisons) compared with 0 μM MMF. *p < 0.01 and ****p < 0.0001 (two-way ANOVA with Tukey’s multiple comparisons) comparing V1 vs V2 at either 10 or 30 μM MMF.

Although we determined MMF induced expression of the OSGIN1-61 kDa isoform in human astrocytes, we could not confirm the isoform at the transcript level. qRT-PCR was unsuccessful because the location of optimized primer/probe sets fell within the overlapping region of *OSGIN1* isoforms. Classical sequencing with PCR using probes generated against the reference sequence was also unsuccessful. Instead, 3′ and 5′ rapid amplification of cDNA ends (RACE) was conducted using RNA extracted from astrocytes treated with a titration of MMF. 3′ RACE probes were designed based on the primer/probe sequences used for *OSGIN1* qRT-PCR. 3′ RACE resulted in an ~1.3 kb sequence confirmed to match the 3′ end of *OSGIN1* following DNA sequencing (data not shown). The identified 3′ RACE product correlated with an increase in total 3′ sequence abundance in the presence of MMF, confirming upregulation of this sequence following MMF treatment (Fig. 5a,b). We then generated primers within the identified 3′ sequenced region for 5′ RACE. 5′ RACE identified a 0.6 kb sequence corresponding to the 5′ end of the *OSGIN1*-52 kDa transcript-encoding region following DNA sequencing (data not shown). The 5′ RACE product was reduced in a dose-dependent manner in the presence of MMF, suggesting the *OSGIN1* 5′ end is differentially regulated in the presence of MMF (Fig. 5a,b).

DNA sequencing of the 0.6 kb 5′ RACE product identified two transcripts corresponding to the 52 kDa OSGIN1 protein (Fig. 5c; V1 vs V2) that differed in two nucleotide substitutions within the 5′ region of *OSGIN1* (Fig. 5c; red letters). Alterations in the 5′ untranslated region (UTR) of *OSGIN1* were previously identified to differentially regulate OSGIN1 protein expression^29^. To determine whether MMF differentially regulates these two transcripts, specific primer/probe sets for each transcript were generated and variant transcripts analyzed in human astrocytes treated with or without MMF. qRT-PCR analysis detected MMF-dependent induction of the V2 variant significantly greater than the V1 variant (*p* = 0.0246, 10 μM MMF; *p* < 0.0001, 30 μM MMF), suggesting they are not simply allelic variants (Fig. 5c,d). The V2 variant substitutes a cytosine (C) for a guanine (G) at position 59 and a G for an adenine (A) at position 62 of the *OSGIN1* sequence (NM_182981.2), resulting in a potential ATG start site and a premature stop site (TGA) (Fig. 5c; underlined letters). These findings suggest that MMF may regulate alterations in the 5′ region of *OSGIN1*; however, further investigation of these alterations is necessary to fully understand their function and contribution to MMF-mediated cytoprotection of astrocytes.

### OSGIN1 induces P53 nuclear translocation, which contributes to MMF-mediated cytoprotection

**Figure 6:**
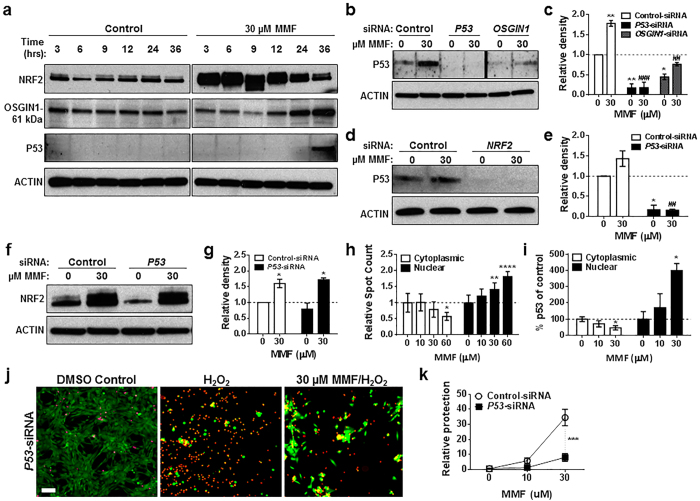
OSGIN1 induces P53 nuclear translocation which contributes to MMF-dependent cytoprotection in human astrocytes. (**a**) Western blot of NRF2, OSGIN1-61 kDa, and P53 from astrocyte extracts following 0 (Control) or 30 μM MMF for 3, 6, 9, 12, 24, or 36 hours. (**b**,**d**) Western blot of P53 from astrocyte extracts following control-, *P53*-, or *OSGIN1*-siRNA (**b**) or control- or *NRF2*-siRNA (**d**) transfection and 0 or 30 μM MMF for 36 hours. Black line in **b** represents blots combined from same experiment/film processed in parallel (Supplementary Fig. S1). (**f**) Western blot of NRF2 from astrocyte extracts following control- or *P53*-siRNA transfection and 0 or 30 μM MMF for 6 hours. ACTIN = loading control (**a**,**b**,**d**,**f**). (**c**,**e**,**g**) Quantification of (**b**,**d**,**f**) using densitometry. Data graphed as relative density to control-siRNA with 0 μM MMF and normalized to 1 (dashed line). Mean ± SEM for *n* = 2–3/condition. *p < 0.05 and **p < 0.01 (two-way ANOVA with Sidak’s multiple comparisons (**c**) or Tukey’s multiple comparisons (**e**,**g**) compared with 0 μM MMF. ^##^*p* < 0.01 and ^###^*p* = 0.0001 (two-way ANOVA with Sidak’s multiple comparisons (**c**) or Tukey’s multiple comparisons (**e**)) compared with 30 μM MMF. (**h**) Quantification of astrocyte nuclear and cytoplasmic P53 following 0, 10, 30, or 60 μM MMF for 36 hours. Data graphed as relative spot count in nuclear or cytoplasmic fractions compared to 0 μM MMF and normalized to 1 (dashed line). Mean ± SD from 6 wells/condition (*n* = 15 fields/well). *p = 0.0169, **p = 0.008 and ****p < 0.0001 (two-way ANOVA with Tukey’s multiple comparisons) compared with 0 μM MMF. (**i**) DNA-binding of astrocyte nuclear or cytoplasmic P53 following 0, 10 or 30 μM MMF. Data graphed as % P53 compared to 0 μM MMF and normalized to 100 (dashed line). Mean ± SD for *n* = 3/condition. *p < 0.05 (two-way ANOVA with Tukey’s multiple comparisons) compared with 0 μM MMF. (**j**) Same as Fig. 3a following control- or *P53*-siRNA transfection. (**k**) Same as Fig. 3b. ***p = 0.0006 compared with control-siRNA with 0 μM MMF (two-way ANOVA with Sidak’s multiple comparisons).

We showed that P53 had no effect on *OSGIN1* transcriptional induction in human astrocytes (Fig. 2c); however, previous research suggests that OSGIN1 and P53 can interact at the protein level within the cytoplasm^20^. To investigate possible changes in P53 protein following MMF administration, human astrocytes were treated with 30 μM MMF for 3, 6, 9, 12, 24, or 36 hours followed by Western immunoblot analysis probed with NRF2, NQO1, OSGIN1, and P53 antibodies (Fig. 6a). MMF induced rapid NRF2 accumulation compared with DMSO control, followed by accumulation of the OSGIN1-61 kDa isoform (Fig. 6a). Although *OSGIN1* transcript levels were found to increase at earlier time points (Fig. 1c), OSGIN1-61 kDa accumulation did not occur until 24 to 36 hours post-MMF treatment (Fig. 6a), suggesting changes in OSGIN1 can occur at either the transcript or protein level eventually leading to protein accumulation post-24 hours. Of particular interest was the observation that MMF induced P53 protein accumulation at 36 hours, which coincided with peak OSGIN1-61 kDa induction (Fig. 6a). To determine whether P53 induction was NRF2- and OSGIN1-dependent, astrocytes were transfected with *P53-, OSGIN1-*, or *NRF2*-siRNA followed by treatment with 30 μM MMF for 36 hours and protein analysis. In the presence of MMF, astrocytes transfected with control-siRNA exhibited a significant P53 induction (*p* = 0.0080) that was abolished with *P53*-siRNA knockdown (*p* = 0.0001) and diminished in astrocytes transfected with *OSGIN1*-siRNA (*p* = 0.0060) (Fig. 6b,c). P53 induction was also lost following *NRF2*-siRNA transfection (*p* = 0.0019) (Fig. 6d,e); however, NRF2 induction in the presence of MMF was maintained following P53 knockdown (Fig. 6f,g). These findings strongly suggest that P53 induction in MMF-treated human astrocytes is NRF2-dependent and occurs downstream of OSGIN1 protein accumulation.

P53 protein levels are tightly regulated within the cell similar to NRF2, and P53 has also been described to be cytoprotective^30^,^31^,^32^. Under resting conditions, P53 is maintained at low levels by proteasomal degradation. Degradation can be inhibited in the presence of ROS resulting in the expression of anti-oxidative stress proteins^33^,^34^. To investigate the effect of MMF on P53 nuclear translocation, human astrocytes were treated with a titration of MMF for 24 hours followed by immunofluorescence microscopy to measure P53 nuclear and cytoplasmic localization using high-content imaging. MMF treatment resulted in significant (*p* = 0.008, 30 μM MMF; *p* < 0.0001, 60 μM MMF) P53 translocation to the nucleus, which correlated with reduced P53 in the cytoplasm (*p* = 0.0169) (Fig. 6h). MMF-induced P53 nuclear translocation in human astrocytes was confirmed using a P53 nuclear TransAM ELISA (Fig. 6i).

Following identification of MMF-dependent P53 nuclear translocation, the importance of P53 in MMF-mediated cytoprotection was evaluated. Human astrocytes were transfected with *P53*- or control-siRNA and treated with 10 or 30 μM MMF for 24 hours followed by oxidative challenge with H_2_O_2_. MMF cytoprotection against H_2_O_2_ insult was diminished in the absence of P53 to a similar extent as observed following *OSGIN1* knockdown (Figs 3e,f and 6j,k). These results suggest that MMF-dependent P53 nuclear translocation may contribute to the cytoprotective properties of MMF in human astrocytes.

### MMF-dependent OSGIN1 expression inhibits cellular proliferation and modulates inflammatory markers

**Figure 7:**
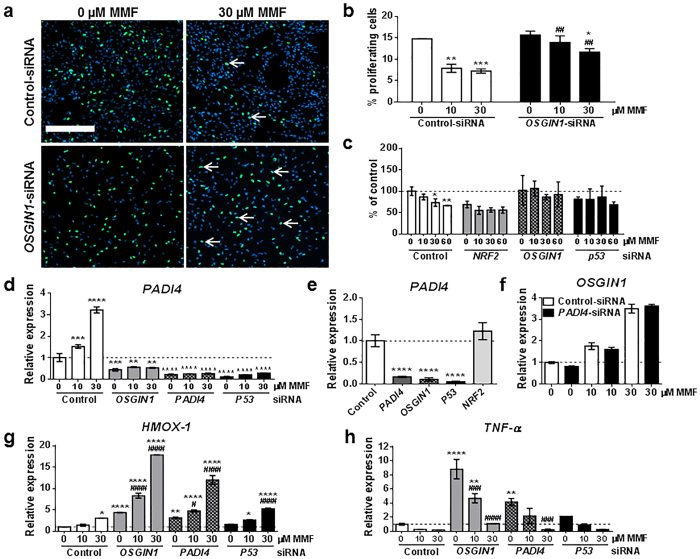
MMF inhibits proliferation of human astrocytes in an OSGIN1-dependent manner. (**a–c**) EdU incorporation in astrocytes following control- (**a**–**c**), *OSGIN1*- (**a**–**c**), *NRF2*- (**c**) or *P53*-siRNA (**c**) transfection and 0, 10, 30, or 60 μM MMF. (**a**) Representative images of **b**: blue (DAPI), green (EdU + cells). Arrows indicate proliferating cells. (**b**) Data graphed as % proliferating cells relative to control-siRNA with 0 μM MMF (mean ± SD from 2 wells (*n* = 15 fields/well/condition). Two-way ANOVA with Sidak’s multiple comparisons (*p < 0.05, **p < 0.01 and ***p < 0.001 (compared with control-siRNA and 0 μM MMF); ^##^*p* < 0.01 (compared with *OSGIN1*-siRNA and 0 μM MMF)). (**c**) Data graphed as % proliferating cells relative to control-siRNA with 0 μM MMF (mean ± SD for *n* = 3/condition) and normalized to 100 (dashed line). *p < 0.05 and **p < 0.01 (one-way ANOVA with Sidak’s multiple comparisons) compared with control and 0 μM MMF. (**d**,**e**) qRT-PCR for *PADI4* following control-, *OSGIN1*-, *PADI4*-, *P53*- or *NRF2*-siRNA transfection and 0, 10, or 30 μM MMF. Data graphed as relative expression to control-siRNA with 0 μM MMF (mean ± SD for n = 2–4/condition) and normalized to 1 (dashed line). (**d**) **p < 0.01, ***p < 0.001 and ****p < 0.0001 (two-way ANOVA) compared with control-siRNA with 0 μM MMF. (**e**) ****p < 0.0001 (one-way ANOVA with Dunnett’s multiple comparisons) compared with control-siRNA. (**f**) qRT-PCR for *OSGIN1* following control- or *PADI4*-siRNA transfection and 0, 10, or 30 μM MMF. Data graphed as relative expression to control-siRNA (mean ± SD for n = 2/condition) and normalized to 1 (dashed line). (**g**,**h**) qRT-PCR for *HMOX1* (**g**) or *TNF-α* (**h**) following control-, *OSGIN1*-, *PADI4*- or *P53*-siRNA transfection and 0, 10, or 30 μM MMF. Mean ± SD (n = 2/condition) graphed as relative expression to control-siRNA and normalized to 1 (dashed line). *p < 0.05, **p < 0.01, and ****p < 0.0001 (two-way ANOVA) compared with control-siRNA and 0 μM MMF. ^#^*p* < 0.05, ^###^*p* < 0.001 and ^####^*p* < 0.0001 (two-way ANOVA) compared with 0 μM MMF for each siRNA transfection.

Regulation of cell cycle is considered a major cellular pathway controlled by OSGIN1^20^,^27^,^35^. To investigate OSGIN1 cell-cycle regulation in CNS-specific cells, human astrocytes were transfected with control- or *OSGIN1*-siRNA followed by 24-hour treatment with MMF. Astrocytes were pulse labeled with 5-ethynyl-2′-deoxy-uridine (EdU) to label dividing cells and analyzed using high-content imaging. MMF administration to cells transfected with control-siRNA significantly reduced total proliferating cells in a dose-dependent manner, whereas MMF had less of an anti-proliferative effect in cells with *OSGIN1*-siRNA knockdown (Fig. 7a,b). This anti-proliferative effect of MMF was diminished following *P53*-siRNA knockdown (Fig. 7c). In contrast to *P53* and *OSGIN1* knockdown, *NRF2* knockdown inhibited overall cellular proliferation with no additional inhibition with MMF (Fig. 7c). Because NRF2 protects cells by various pathways, loss of NRF2 could place transfected cells in a high state of stress, making them increasingly sensitive to their environment. Overall, these results suggest that inhibition of cellular proliferation in the presence of MMF is OSGIN1- and P53-dependent.

P53 is cited in the literature as a regulator of *OSGIN1* transcript expression in immortalized/transformed cells^20^; however, our results in human astrocytes suggest OSGIN1 regulates nuclear translocation of P53 protein. Peptidyl arginine deiminase type IV (PADI4) is cited to negatively regulate *OSGIN1* expression^26^ and to induce gene transcription by regulating the deimination of arginine residues on histones and antagonizing arginine methylation, which may contribute to cell-cycle control^36^. To determine whether *PADI4* is transcriptionally regulated following MMF treatment, human astrocytes transfected with control-, *OSGIN1*-, *PADI4*-, or *P53*-siRNA were analyzed for *PADI4* mRNA expression following MMF treatment. MMF treatment of control siRNA-transfected cells significantly induced *PADI4* mRNA expression (*p* < 0.001), which was abolished in cells transfected with *OSGIN1*-, *PADI4*- or *P53*-siRNA (*p* < 0.0001) (Fig. 7d). Furthermore, *PADI4* induction was independent of NRF2 (Fig. 7e). Knockdown of PADI4 also had no effect on *OSGIN1* mRNA levels in the presence of MMF, suggesting PADI4 does not negatively regulate *OSGIN1* levels in astrocytes (Fig. 7f). Based on the known function of PADI4 as a mediator of cell proliferation, PADI4 may inhibit cell division in the presence of MMF in a P53-dependent manner; however, further investigation is necessary to fully understand the role of PADI4 in human astrocytes following MMF treatment.

Along with mediating cell cycle, OSGIN1 has been shown to regulate inflammation^37^. To determine whether OSGIN1 regulates transcripts encoding for inflammatory markers, *OSGIN1-, PADI4-*, and *P53-*siRNA knockdown was analyzed for *HMOX-1* and tumor necrosis factor alpha (*TNF-α*) mRNA via qRT-PCR following MMF treatment in human astrocytes. Cells treated with DMSO, *OSGIN1*- and *PADI4*-siRNA, significantly induced *HMOX-1* transcript expression compared with control-siRNA (*p* < 0.01; asterisks), suggesting OSGIN1 and PADI4 typically repress some level of *HMOX-1* expression (Fig. 7g). No change in *HMOX-1* was detected in cells treated with DMSO following P53 knockdown (Fig. 7g). *HMOX-1* mRNA levels were increased to a significantly greater extent with 10 and 30 μM MMF treatment in *OSGIN1*- and *PADI4*-siRNA transfected cells, whereas *HMOX-1* mRNA levels were increased to a lesser extent and only with 30 μM of MMF treatment in control- and *P53*-siRNA transfected cells (Fig. 7g). When analyzed for *TNF-α* expression, cells transfected with *OSGIN1*-, *PADI4*-, and *P53*-siRNA had significantly greater *TNF-α* induction compared with control-siRNA (*p* < 0.02; asterisks) (Fig. 7h). However, MMF administration reduced *TNF-α* mRNA across all transfected groups (Fig. 7h). These findings suggest that OSGIN1, PADI4, and P53 may modulate inflammatory processes both in the presence and absence of MMF.

## Discussion

NRF2 activation by small-molecule compounds such as DMF increases expression of antioxidant-defense genes, which promotes cytoprotection in various models of neurodegenerative disease^12^,^13^. However, the exact mechanisms underlying NRF2-mediated cytoprotection are unclear. We previously conducted transcriptional profiling studies to evaluate gene regulation following oral DMF administration to identify CNS-specific targets. The NRF2 transcriptional target *Osgin1* was the most robustly modulated transcript in brain^17^. Current literature describes OSGIN1 as a major mediator of cellular apoptosis under the control of the tumor suppressor protein, P53^26^. However, our findings in primary human astrocyte cultures suggest *OSGIN1* is a transcriptional target of NRF2, not P53. *OSGIN1* modulates P53 protein and contributes to the cytoprotective properties of MMF, the bioactive metabolite of DMF.

After observing that MMF-induced *OSGIN1* expression was NRF2-dependent, rather than P53-dependent, the next question was whether *OSGIN1* splice variants contribute to alternative mechanisms under NRF2 transcriptional control. Most studies on OSGIN1 function used tumor cell lines and identified the shorter OSGIN1-52 kDa isoform as a strong apoptotic inducer under the P53 control^21^,^28^. In contrast, the longer OSGIN1-61 kDa isoform is not shown to strongly induce apoptosis in tumor cell lines. We identified the OSGIN1-61 kDa isoform, but not the OSGIN1-52 kDa isoform, to be regulated in human astrocytes following MMF treatment in an NRF2-dependent manner. The specific, cytoprotective regulation of OSGIN1-61 kDa under the transcriptional control of NRF2 supports an alternative mechanism for OSGIN1 independent of the recognized function of this gene in tumor cell lines. These findings suggest functional domains within OSGIN1 isoforms may result in differing biological effects regulated under specific cellular conditions which may explain how OSGIN1 contributes to diverse cellular functions in addition to apoptosis, such as anti-inflammatory actions, regulation of cell-cycle, and protection against oxidative stress^18^,^38^. We also identified a role for OSGIN1 in regulating anti-inflammatory effects based on increased expression of *TNF-α* and *HMOX-1* following *OSGIN1*-siRNA knockdown in astrocytes. This correlates with previous findings that OSGIN1 can reduce oxidative stress and inflammation in cells challenged with oxidized 1-palmitoyl-2-arachidonoyl-sn-glycero-3-phosphocholine^18^,^37^. Overall, our results support a protective role for the OSGIN1-61 kDa isoform in human astrocytes against oxidative and inflammatory stress.

We used several methods to determine how MMF regulates specific *OSGIN1* transcripts in human astrocytes. Although the specific transcript encoding the identified OSGIN1-61 kDa protein product was not detected, a distinct reduction in expression of the 5′ end of the MMF-induced transcript was identified in RACE analysis. DNA sequencing determined the MMF-induced transcript to encode for the OSGIN1-52 kDa isoform, suggesting changes in this isoform may also occur following MMF treatment. The inability to properly sequence the *OSGIN1*-61 kDa isoform may be a result of GC enrichment in the 5′ region or be a result of the low abundance of this transcript, resulting in an inability to fully sequence the long *OSGIN1* isoform. In general, these findings suggest that alterations in 5′ splicing do occur in the presence of MMF. Alternate splicing of *OSGIN1* was further supported by identification of two nucleotide substitutions in the 5′ end of the identified transcript induced following MMF treatment in astrocytes, which resulted in a potential AUG start site encoding a small open reading frame (ORF). Although the Kozak region preceding this new start site was not strong, evidence from current literature suggests upstream AUG sites encoding upstream ORFs (uORFs) in 5′ UTR regions can decrease the frequency of downstream AUG start sites to initiate transcription in the main ORF^39^. Furthermore, the generation of *OSGIN1* small encoding ORFs in the 5′ UTR, have been previously identified to negatively control OSGIN1 protein translation^29^. Therefore, the regulation of specific *OSGIN1* transcripts by MMF and NRF2 could potentially result in expression bias for the 61 kDa protein over the 52 kDa form through downregulation of OSGIN1-52 kDa protein expression independent of transcript regulation. Preliminary studies in our lab show evidence of reduced OSGIN1-52 kDa protein expression in the presence of MMF; however, further experiments are necessary to thoroughly investigate this hypothesis.

As mentioned, P53 did not regulate *OSGIN1* expression in human astrocytes. Instead, treatment of cells with MMF identified P53 to be a protein target downstream of NRF2-regulated *OSGIN1*. The inability of P53 to accumulate and translocate to the nucleus in the absence of OSGIN1, suggests OSGIN1 may activate P53-mediated transcriptional control. Previous studies have suggested that P53 and NRF2 work together to regulate gene expression in the presence of oxidative stress and P53 itself has also been shown to be protective against oxidative damage^31^. Thus, *OSGIN1* activation by NRF2 may be a potential mechanism through which NRF2 regulates P53 during periods of oxidative stress. Because *OSGIN1* gene expression is activated prior to classical NRF2 targets, OSGIN1 may function to induce P53 translocation to the nucleus where it can interact with NRF2 to regulate other NRF2 target genes. Data from other labs have already identified the ability of P53 and OSGIN1 to interact in the cytoplasm, but there may also be a role for OSGIN1 in inhibiting degradation of P53 similar to the degradation mechanism of NRF2. One way to investigate these possibilities would be to conduct P53 pull-down studies to measure the interaction of NRF2 and OSGIN1 with P53. Furthermore, investigation into the interaction of OSGIN1 with the protein degradation machinery associated with P53 could give insight into whether or not OSGIN1 inhibits P53 degradation.

The translocation of P53 to the nucleus by OSGIN1 may also induce or suppress NRF2-independent gene regulation. This was supported by our evidence showing regulation of *PADI4* by P53 in astrocytes was NRF2-independent. We also identified *PADI4* to be transcriptionally regulated by OSGIN1, suggesting that OSGIN1-mediated translocation of P53 induces *PADI4* expression. Similar to P53, the regulation of *PADI4* in association with OSGIN1 contrasts with current literature that suggests PADI4 to be a negative regulator of *OSGIN1*^26^. The alternate functions of OSGIN1 in astrocytes described above may be fumarate-specific because expression of the OSGIN1-61 kDa isoform and cell type-specific signaling of OSGIN1 in astrocytes was similar with DMF (data not shown). PADI4 has been shown to regulate transcription through DNA methylation by citrullination of histone residues, and a role for P53 in this process has been described^36^. Therefore, in human astrocytes, PADI4 may inhibit DNA transcription in a P53-dependent manner to reduce cell proliferation, a process that is independent of apoptosis based on our findings that MMF does not induce apoptosis, but instead reduces cell proliferation. Various studies have identified the inhibition of cell-cycle entry to be a protective mechanism allowing cells to conserve energy and limit the replication of DNA damage^40^,^41^. Based on the known function of PADI4 as mediator of cell proliferation, PADI4 may function to inhibit cell division in the presence of MMF in a P53-dependent manner. Contribution of PADI4 to cellular protection in the presence of MMF is currently being investigated.

**Figure 8:**
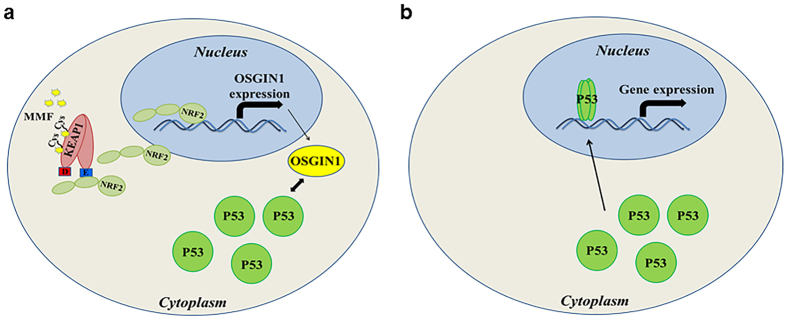
Potential mechanism of action of OSGIN1-mediated cellular protection. (**a**) Interaction of MMF with cysteine residues on KEAP1 results in an allosteric conformational change in the KEAP1 protein so that NRF2 is no longer targeted for ubiquitination and proteasomal degradation. This allows NRF2 to accumulate in the cytoplasm and translocate to the nucleus where it can regulate the transcription of various genes including, *OSGIN1. OSGIN1* transcript expression is then translated to a 61 kDa protein that can induce the accumulation of P53 by an unknown mechanism. (**b**) P53 protein induced by OSGIN1-61 kDa can then translocate to the nucleus and induce gene transcription.

NRF2-mediated cytoprotection is not simply a result of antioxidant induction, but instead consists of a complicated network of pathways that function together to protect cells during periods of stress. Overall, our results indicate a mechanism for NRF2-dependent transcription of *OSGIN1* in MMF-mediated cytoprotection against oxidative stress (Fig. 8). *OSGIN1*-mediated cytoprotection involves NRF2 activation by MMF through interaction of KEAP1 cysteines, resulting in inhibition of NRF2 degradation and subsequent NRF2 nuclear translocation (Fig. 8a). Inside the nucleus, NRF2 regulates cytoprotective gene transcription including *OSGIN1*, one of the earliest NRF2-transcribed targets. Translation of the OSGIN1-61 kDa protein results in the accumulation and subsequent translocation of P53 to the nucleus, leading to additional target gene induction and potentially inhibition of cell proliferation (Fig. 8b). This series of events suggests a mechanism by which NRF2, OSGIN1, and P53 cooperate to protect cells against oxidative insult in the presence of MMF. Many aspects of this theoretical pathway of MMF-induced cellular protection need further investigation, such as ref. 1 whether P53 regulates genes independently or in collaboration with NRF2^2^, understanding the importance of inhibiting cell-cycle and proliferation in this paradigm when our evidence suggests this to be a protective mechanism including^3^, possibly controlling inflammatory responses given our observation that loss of *OSGIN1* increased *TNF-α* levels. This study provides mechanistic insight into MMF-mediated cytoprotection via NRF2, OSGIN1, and P53 in CNS-derived cells. Understanding the contribution of specific NRF2 target genes to cytoprotection in diverse cell types can assist in developing therapeutics to modulate this pathway in various diseases.

## Methods

### Cell Culture

Primary cultures of human spinal cord astrocytes were purchased from ScienCell Research Laboratories (Carlsbad, CA), grown in Astrocyte Medium (ScienCell) and maintained according to supplier specifications. Cells for plate-based assays were seeded into poly-D-lysine tissue culture 24- or 96-well plates (BD Biosciences, San Jose, CA).

### Compound Handling

MMF was prepared at 100 mM in dimethyl sulfoxide (DMSO), titrated in DMSO, and diluted into Astrocyte Medium for cell treatments. The final concentration of DMSO (0.03%) was consistent for all treated cells.

### Cellular Extract Preparation, NRF2 and P53 Activity Assays, and Western Blotting

Cytosolic and nuclear extracts were prepared using a nuclear extract kit from Active Motif Inc. (Carlsbad, CA). Whole-cell extracts for Western blotting were collected directly in 1X Laemmli denaturing buffer (63 mM Tris HCl, 10% glycerol, 2% sodium dodecyl sulfate, 0.01% Bromophenol Blue, 2% beta-mercaptoethanol, pH 6.8). Protein was quantified using the Pierce^®^ BCA Protein Assay Kit (ThermoFisher, Waltham, MA) and samples were diluted to equal loading volumes. Antibodies for Western blotting and the TransAM NRF2 and P53 assays (Active Motif Inc.) were used according to the manufacturer’s instructions. The following antibodies were used at 1:1000: NRF2 (Abcam, Cambridge, UK), NQO1 (Abcam), P53 (Cell Signaling, Danvers, MA) and HDAC1 (Cell Signaling). ACTIN antibody (MP Biomedicals, Solon, OH) was used at 1:5000. Immunoblots were quantitated by densitometry.

### Quantitative Real-Time Polymerase Chain Reaction

Total mRNA extraction and quantitative real-time polymerase chain reaction (qRT-PCR) were performed as previously described^16^. All TaqMan^®^ Gene Expression Assays were purchased from Life Technologies (Grand Island, NY) at a concentration of 20X and included the following: *NQO1*, Hs02512143_s1; *OSGIN1*, Hs00203539_m1; *NRF2*, Hs00975961_g1; *P53*, Hs01034249_m1; *PADI4*, Hs00202612_m1; *HMOX-1*, Hs01110250_m1; *TNF-α*, Hs01113624_g1; and *ACTIN*, Hs01060665_g1. Custom primers for *OSGIN1* 5′ UTR analysis were generated using primer express software and analyzed using Basic Logical Alignment Search Tool (BLAST) to confirm specificity. Custom *OSGIN1* primer/probe sets included: V1 [forward primer (CTTCCCTCTGGCCTCTCAGA), reverse primer (GAGATCGGGACACCCATTACC) and probe (CCTCTTGGATCCCC)] and V2 [forward primer (AATGGGTGTCCCGATGTCA), reverse primer (CCGGCCAAGTTGTGCACTA) and probe (ACTCTGTGATCCGTGTTC)]. All samples were measured in duplicate or triplicate using *ACTIN* as a normalizing gene. Final analysis used the comparative CT method to calculate fold changes and samples were normalized relative to vehicle or DMSO control conditions within each data set.

### Small Interfering RNA Transfection

Human spinal cord astrocytes were transfected with small-interfering RNA (siRNA) using X-tremeGENE siRNA transfection reagent (Roche, Indianapolis, IN) and the reverse transfection method^42^. All constructs were purchased from OriGene technologies (Rockville, MD) and cells were transfected with 10 nM siRNA targeted against *NRF2*-, *OSGIN1*-, *NQO1*-, *P53*-, *PADI4*- or nonspecific-siRNA. Cells were incubated for 12 hours after transfection followed by replacement of Astrocyte Media. Knockdown was assessed at 48 hours post-transfection by qRT-PCR and Western blotting. For analysis with MMF, cells were treated with a titration of MMF 24 hours post-transfection and analyzed in plate-based assays as described below.

### Generation of OSGIN1 isoform-specific antibodies

Three isoform specific rabbit polyclonal antibodies were generated by New England Peptide (NEP, Gardner, MA) based on the sequences identified and cloned by Ong *et al*.^21^. Peptides were generated against human specific sequences in the OSGIN1-38 kDa, −52 kDa, and −61 kDa protein regions, although the 38 kDa isoform is not currently accepted as a likely variant. Based on the methods of NEP, antibodies were generated and affinity purified using OSGIN1 isoform-specific peptides. Due to the overlapping domains of the sequences, only the 61 kDa peptide sequence resulted in an isoform-specific antibody. Peptide competition confirmed antibody specificity for both the OSGIN1-52 kDa and −61 kDa reactive antibodies. No immunoreactive bands were identified with the OSGIN1-38 kDa antibody (data not shown) and hence was not furthered pursued.

### Immunostaining

Immunostaining was conducted as previously described^16^. Primary antibodies (P53, Cell Signaling; OSGIN1, NEP) and secondary antibodies (Alexa Fluor^®^ 488, ThermoFisher) were used according to the manufacturer’s instructions. Nuclei were labeled with 4′,6-diamidino-2-phenylindole, dihydrochloride (DAPI, ThermoFisher). Images were acquired using the Thermo HCS Arrayscan VTI platform (ThermoFisher) with a modified algorithm to measure total DAPI count, P53 nuclear versus cytoplasmic signal, and total OSGIN1 positive puncta.

### Plate-Based Cellular Assays

Twenty-four hours post-transfection or plating, human spinal cord astrocytes were treated with 0, 10, or 30 μM of MMF for 20 hours followed by a 2-hour challenge with 0, 200, or 300 μM hydrogen peroxide (H_2_O_2_) diluted in Hank’s balanced salt solution (plus 20 mM HEPES, pH 7.4). Cells were allowed to recover for 20 hours and cellular viability was assessed using a LIVE/DEAD viability stain (ThermoFisher) according to the manufacturer’s protocol. Viable cells were quantified by fluorescence intensity from LIVE stain (calcein AM; excitation wavelength, 488 nM; emission wavelength, 525 nM) and in parallel by counting DAPI-labeled nuclei. For proliferation assays, cells were incubated with 5-ethynyl-2′-deoxyuridine (EdU) and proliferating cells were counted according to the manufacturer’s protocol outlined for the Click-iT^®^ EdU HCS Assay (ThermoFisher). EdU was added to cells at a 1000-fold dilution in Astrocyte Media and pulse labeled for 1 hour. Following EdU incorporation, cells were fixed in 4% paraformaldehyde (PFA)/4% sucrose in PBS, and immunostained plates quantitated for EdU incorporation. Cellular apoptosis was assessed in fixed cells (4% PFA/4% sucrose) using the HT TiterTACS™ Assay Kit (Trevigen, Gaithersburg, MD) according to the manufacturer’s protocol. LIVE/DEAD, Click-IT^®^ EdU, and TiterTACS™ assays were analyzed using automated imaging and counting with the ThermoFisher HCS Arrayscan VTI platform and associated algorithm creation.

### Rapid Amplification of cDNA Ends

All primers for RACE were generated against the human *OSGIN1* sequence and analyzed using BLAST to confirm specificity. 3′ RACE primers included: gene-specific primer 1 (GSP1, GCTCCCGGACCTGGAGGT) and nested-GSP2 (ACTGGATGCAGAAGAAGCGA). 5′ RACE primers included: GSP1 (CGCTTCTTCTGCATCCAGTCC), GSP2 (GCATCCAGTCCTTGACCTCCA), and nested-GSP3 (TGACCTCCAGGTCCGGGAGC). RACE was conducted using 3′ and 5′ RACE Kits (ThermoFisher). The manufacturer’s protocol was followed for RACE other than for target cDNA amplification, which was done using Platinum PCR Supermix (ThermoFisher). 3′ RACE products were separated by gel electrophoresis, excised, and inserted into a TOPO vector according to the manufacturer’s protocol (ThermoFisher). The vector-DNA ligation was then transformed into One Shot^®^ Chemically Competent *E. coli* (ThermoFisher) and DNA purified according to the manufacturer’s instructions. DNA was checked by restriction digest followed by DNA sequencing.

## Additional Information

**How to cite this article**: Brennan, M. S. *et al*. The NRF2 transcriptional target, *OSGIN1*, contributes to monomethyl fumarate-mediated cytoprotection in human astrocytes. *Sci. Rep.* **7**, 42054; doi: 10.1038/srep42054 (2017).

**Publisher's note:** Springer Nature remains neutral with regard to jurisdictional claims in published maps and institutional affiliations.

## Supplementary Material

Supplemental Figure S1
