# Supplementary material for: The NRF2 transcriptional target, OSGIN1, contributes to monomethyl fumarate-mediated cytoprotection in human astrocytes
Source: Sci Rep. 2017 Feb 9;7:42054. doi: 10.1038/srep42054 (PMC5299414; doi:10.1038/srep42054)

**The NRF2 transcriptional target, *OSGIN1*, contributes to monomethyl fumarate-mediated cytoprotection in human astrocytes**

*Melanie S. Brennan^1,2^, Maria F. Matos^1^, Karl E. Richter^1^, Bing Li^1^ and Robert H. Scannevin^1^

*^1^ Neurology Research, Biogen Inc., Cambridge, MA, 02142*

*^2^ Boston University School of Medicine, Boston, MA 02118*

**Supplemental Figure Legend**

**Supplemental Figure S1.** Uncropped images from Figure 6b. (**a**,**b**) Two blots from the same gel, derived from the same experiment and processed in parallel were combined for simplicity in Figure **6b**. Black boxes represent the blot regions combined for use in Figure **6b** to represent P53 protein expression. Blots labeled “separate antibody” represent blots from the same gel probed with a non-P53 antibody. **b** is a lower exposure of **a**.


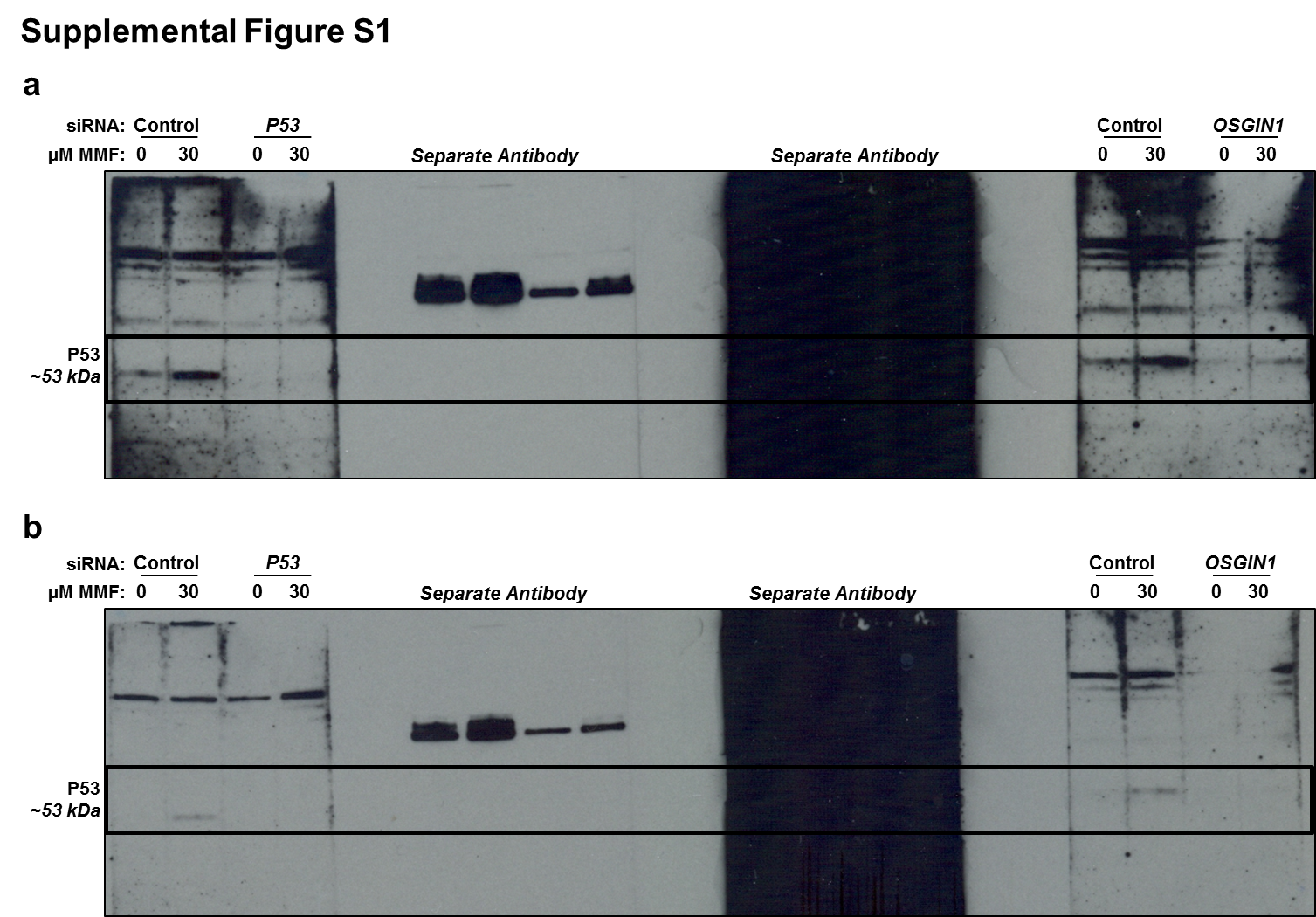

Supplement: Supplemental Figure S1 [file srep42054-s1.doc]
